# Association of Mu-Opioid Receptor Expression With Long-Term Survival and Perineural Nerve Invasion in Patients Undergoing Surgery for Ovarian Cancer

**DOI:** 10.3389/fonc.2022.927262

**Published:** 2022-07-07

**Authors:** Hao Zhang, Mengdi Qu, Caihong Sun, Yanghanzhao Wang, Ting Li, Wei Xu, Zhirong Sun, Xiaoguang Zhang, Kefang Guo, Wankun Chen, Minli Sun, Changhong Miao

**Affiliations:** ^1^ Department of Anesthesiology, Zhongshan Hospital, Fudan University, Shanghai, China; ^2^ Shanghai Key Laboratory of Perioperative Stress and Protection, Shanghai, China; ^3^ Department of Anesthesiology, Jinshan Hospital, Fudan University, Shanghai, China; ^4^ Department of Anesthesiology, Fudan University Shanghai Cancer Center, Shanghai, China; ^5^ Department of Oncology, Shanghai Medical College, Fudan University, Shanghai, China

**Keywords:** ovarian cancer, mu-opioid receptor, overall survival, disease-free survival, surgery

## Abstract

**Background:**

Opioids are widely used during primary debulking surgery (PDS) for ovarian cancers, and a high mu-opioid receptor (MOR) expression predicts worse cancer outcomes. However, the impact of MOR expression on survival outcomes in ovarian cancers is still not clear.

**Methods:**

A retrospective cohort study was conducted in patients who underwent PDS in ovarian cancer patients. MOR expression was measured in tumor and normal tissue. Primary outcomes were overall survival (OS) and disease-free survival (DFS). Secondary outcomes included perineural invasion (PNI), intraoperative sufentanil consumption, length of stay (LOS), and verbal numerical rating scale (VNRS) on postoperative day 1 (POD1), POD3, and POD5.

**Results:**

After propensity score matching, a total of 366 patients were finally enrolled in this study. There were no significant differences in OS rates in patients with high versus low levels of MOR (1-year OS: 82.9% versus 83.3%, 3-year: 57.8% versus 59.1%, 5-year: 22.4% versus 23.1%,respectively) in the ovarian cancers. There were no significant differences in DFS between the groups. Intraoperative sufentanil consumption was higher in the MOR high-expression group compared with the MOR low-expression group. Tumors expressing high levels of MOR showed higher rates of PNI. VNRS in the MOR high-expression group was higher on POD1.

**Conclusion:**

MOR is not an independent predictor of worse survival in ovarian cancers but is associated with high rates of perineural invasion.

## Introduction

Ovarian cancer is the third most common gynecological tumor and ranks 5th in all cancer-related deaths in women (1). Although significant progress has been made in the early diagnosis and treatment of ovarian cancer in recent years, the 5-year survival rate of ovarian cancer patients is still lower than 40% (2). This worrisome statistics highlights the need for new therapies.

Primary debulking surgery (PDS) remains the cornerstone in ovarian cancer treatment (3). Primary ovarian cancer surgery is performed to achieve optimal cytoreduction, as the amount of residual tumor is one of the most important prognostic factors for survival of women with high-stage epithelial ovarian cancer (3). Opioids remain the primary analgesics during and after ovarian cancer surgery (4, 5). Opioids mainly exert their analgesic effect by acting as agonists of the mu-opioid receptor (MOR) located in neurons, but it is also expressed on cancer cells (5–7). Previous clinical studies have found that a high tumoral MOR expression is associated with poor prognosis in hepatocellular, laryngeal, and lung cancers (8–10). Furthermore, MOR expression was associated with high perineural nerve invasion (PNI), a clinical predictor of survival in pancreatic and laryngeal cancers (9, 11). In contrast, other studies have found that MOR expression is not a predictor of worse long-term survival in pancreatic and colorectal cancers (11–13).

The association between MOR expression and the long-term prognosis of ovarian cancer is still unclear. Therefore, we conducted a retrospective study and hypothesized that a high expression of MOR is associated with poor prognosis in ovarian cancer. In addition, we determined the impact of MOR expression on length of hospital stay (LOS), intraoperative opioid consumption, and postoperative pain intensity.

## Methods

### Study Population

This study was conducted at the Fudan University-affiliated hospitals and obtained ethics committee board approval. The inclusion criteria for this study were a) women undergoing PDS for ovarian cancer from January 2015 to December 2018, PDS criteria based on International Federation of Gynecology and Obstetrics (FIGO) stage III or IV ovarian, tubal, and peritoneal cancers diagnosed using clinical findings, including imaging studies (CT, MRI, and chest radiography) and cytology of ascites, pleural effusions, or tumor cyst fluids obtained by tumor centesis; b) aged between 18 and 70 years; c) undergoing surgery under combined general and epidural anesthesia; and d) complete clinical characteristics and follow-up data. Patients were excluded if they met the following exclusion criteria: a) underwent second-time or emergency surgery; b) had a history of other malignancies; c) died within hospital stay after surgery; and d) lost to follow-up. We define surgical complexity based on the number and complexity of the surgical procedures performed. Scores ranging from 1 to 3 were assigned to each surgical procedure based on the complexity of the procedure. We then developed an ordinal scale so that the patients could be stratified into three groups: simple, intermediate, and complex surgery (14).

### Co-Primary Outcomes

The primary outcomes of this study were overall survival (OS) and disease-free survival (DFS). OS was defined from the surgery date to the date of death or last lost follow-up (15). DFS was determined from the surgery date to the date of ovarian cancer recurrence (15). Routine clinical follow-ups were done every 3 months in the first and second years and every 6 months in the third to fifth years. The final follow-up date was January 31, 2020. Cancer recurrence was determined using a combination of computed tomography scan, positron emitted tomography scan, and serum concentrations of CA-125 (16).

### Secondary Outcomes

Secondary outcomes included PNI, length of stay, intraoperative sufentanil consumption, and pain intensity using the verbal numeric rating scale (0: no pain–10: worst pain ever).

### Anesthesia Care

All patients were monitored according to American Society of Anesthesiologists (ASA) guidelines. Induction of general anesthesia was performed with propofol (3.0–4.0 µg/ml, target-controlled infusion protocol (TCI)), sufentanil (0.3–0.5 µg/kg), and rocuronium (o.5 mg/kg). After induction of general anesthesia, patients were tracheal intubated, and general anesthesia was maintained with 2.0%–3.0% sevoflurane in a mixture of oxygen/air. An epidural infusion of 0.375% ropivacaine was used during surgery. After surgery, patients received patient-controlled epidural analgesia (PCEA, 0.1% ropivacaine and 0.5 µg/ml sufentanil, basal infusion: 2–3 ml/h, bolus: 3–4 ml, lockout time: 15 min) for 48 h.

### Immunohistochemistry and PNI

All the samples were retrieved from banked tissue samples. Briefly, immunohistochemistry (IHC) staining was performed in ovarian tumor or normal tissue (ovarian). The primary antibody was the anti-mu opioid receptor (UMB3) C-terminal (ab134054). The antibody was used at a concentration of 1:200. Secondary antibodies anti-Goat Anti-Rabbit IgG H&L (HRP) (ab205718) were used. After staining, two pathologists blinded to clinical data reviewed and scored the sections independently. The IHC score was calculated as previously reported (11). Briefly, the intensity of MOR was graded from 0 to 3, and the percentage of MOR positive was also graded from 0 to 3 (score 0: <25% positive, score 1: 25%–50% positive, score 2: 51%–75% positive, and score 3: >75% positive). A total score from 0 to 6 was calculated (11). PNI was defined as cancer cells that invade the perineural spaces of surrounding nerves (17).

### Statistical Analysis

Patients’ characteristics were summarized with descriptive statistics. Continuous data were expressed with mean ± standard deviation (SD) and analyzed with a t-test. Categorical data were described with n (%) and analyzed with the chi-square test. Chi-square or Fisher’s test was used to evaluate associations between categorical variables. The Mann–Whitney U test or t-test was used to assess continuous variables between the groups. The Kaplan–Meier method was used to analyze OS and DFS in the model. Hazard ratios (HR) were calculated with corresponding 95% confidence intervals (CI). Multivariable Cox proportional hazard models were used, including significant covariates. From a recent retrospective study in a similar population of patients (3), the median overall survival time of subjects was 42.3 and 38.5 months, respectively. Assuming that alpha = 0.05, with a two-sided test having power of 80%, a total of 583 participants would be required to detect a 3.8-month difference in overall survival between groups. Because we anticipated a dropout rate of 8%, we planned to enroll 633 patients in the trial. We performed propensity score matching to reduce bias using a 5- to 1-digit Greedy matching algorithm (3). Ten variables were used in the model, including age, body mass index (BMI), ASA class, Charlson comorbidity index (CCI), histologic diagnosis, tumor differentiation, surgical complexity, residual disease, and adjuvant chemotherapy. The standardized differences for all covariates did not exceed 3.45% in the post-matching cohort, suggesting a substantial reduction of bias between the two groups. The mean cutoff values for MOR expression were analyzed with X-Tile software (17). A P-value <0.05 was considered statistically significant. Statistical analyses were performed with SPSS 17.0 (SPSS Inc., Chicago, IL, USA).

## Results

A total of 483 patients were included in the study. After the initial examination, 206 patients were grouped in the high MOR expression cohort and 277 in the low-expression group. After propensity score matching, 183 patients remained in each group (MOR high versus MOR low). The baseline characteristics were similar between both groups of patients (**Table 1**).

**Table 1:** Patient and treatment characteristics for both groups.

Variable	Original cohort		*P*	Matched cohort		*P*	Standard difference (%)
	MOR high expression (n = 206)	MOR low expression (n = 277)		MOR high expression (n = 183)	MOR low expression (n = 183)		
**Age (years)**	53.6 ± 8.6	54.2 ± 8.2	0.436	53.2 ± 10.2	53.4 ± 10.6	0.854	1.08
**BMI (kg/m^2^)**	25.6 ± 6.3	26.3 ± 6.2	0.224	25.3 ± 6.2	26.4 ± 6.3	0.093	1.65
**ASA (n, %)**			0.857			0.808	2.23
I–II	151 (73.2%)	201 (72.6%)		137 (75.1%)	139 (75.8%)		
III–IV	55 (26.8%)	76 (27.4%)		46 (24.9%)	44 (24.2%)		
**Patients enrolled**			1.000			0.775	
2015	49 (23.7%)	65 (23.5%)		42 (23.1%)	43 (23.5%)		
2016	46 (22.5%)	63 (23.1%)		41 (22.8%)	42 (23.2%)		
2017	50 (24.3%)	67 (24.2%)		45 (24.8%)	47 (25.5%)		
2018	61 (29.5%)	82 (29.2%)		55 (29.3%)	51 (27.8%)		
**CCI (n, %)**			0.667			0.976	3.35
0	36 (17.5%)	46 (16.8%)		32 (17.8%)	31 (17.3%)		
1	90 (43.7%)	123 (44.5%)		78 (42.6%)	77 (41.9%)		
≧2	80 (38.8%)	108 (38.7%)		73 (39.6%)	75 (40.8%)		
**Histologic diagnosis**			0.880			0.745	2.14
Serous histology	131 (63.6%)	178 (64.3%)		114 (62.5%)	117 (63.9%)		
Non-serous histology	75 (36.4%)	99 (35.7%)		69 (37.5%)	66 (36.1%)		
**Tumor size**			0.830			0.816	1.96
>5	121 (58.9%)	160 (57.8%)		106 (58.1%)	105 (57.4%)		
<5	85 (41.1%)	117 (42.2%)		77 (41.9%)	78 (42.6%)		
**Tumor differentiation**			0.038			0.575	3.45
Well	19 (9.3%)	26 (9.5%)		17 (9.4%)	17 (9.3%)		
Moderate	116 (56.3%)	164 (59.2%)		99 (54.1%)	97 (53.5%)		
Poor	71 (34.4%)	87 (31.3%)		67 (36.5%)	69 (37.2%)		
**Residual disease**			0.550			0.865	3.24
No visible disease	98 (47.4%)	129 (46.7%)		83 (45.6%)	85 (46.3%)		
<1-cm residual disease	70 (34.1%)	98 (35.4%)		64 (35.4%)	66 (36.3%)		
>1-cm residual disease	38 (18.5%)	50 (17.9%)		36 (19%)	32 (17.4%)		
**Surgical complexity**			0.855			0.873	
Low	31 (15.4%)	45 (16.2%)		26 (14.2%)	26 (14.3%)		
Intermediate	108 (52.6%)	148 (53.6%)		98 (53.6%)	96 (52.7%)		
High	67 (32%)	84 (30.2%)		59 (32.2%)	61 (33%)		
**Surgery time (min)**	213 ± 63	209 ± 59	0.474	205 ± 61	208 ± 62	0.641	
**Ascites (ml)**			0.495			0.849	
<200	36 (17.5%)	51 (18.3%)		29 (15.9%)	28 (15.6%)		
>200	29 (14.1%)	41 (14.8%)		26 (14.2%)	27 (14.5%)		
**Estimated blood loss (n, %)**			0.750			0.716	
≤400 ml	116 (56.3%)	160 (57.7%)		101 (55.4%)	100 (54.7%)		
>400 ml	90 (43.7%)	117 (42.3%)		82 (44.6%)	83 (45.3%)		
**Blood transfusion**			0.798			0.615	
No	131 (63.6%)	173 (62.5%)		112 (61.3%)	111 (60.5%)		
Yes	75 (36.4%)	104 (37.5%)		71 (38.7%)	72 (39.5%)		
**Adjuvant Chemotherapy (n, %)**			0.487			0.811	3.36
No	63 (30.4%)	93 (33.5%)		59 (32.1%)	60 (32.6%)		
Yes	143 (69.6%)	184 (66.5%)		124 (67.9%)	123 (67.4%)		

BMI, body mass index; ASA, American Society of Anesthesiologists score; CCI, Charlson Comorbidity Index; FIGO, Federation International of Gynecology and Obstetrics.

### Primary Outcome

**Figure 1:**
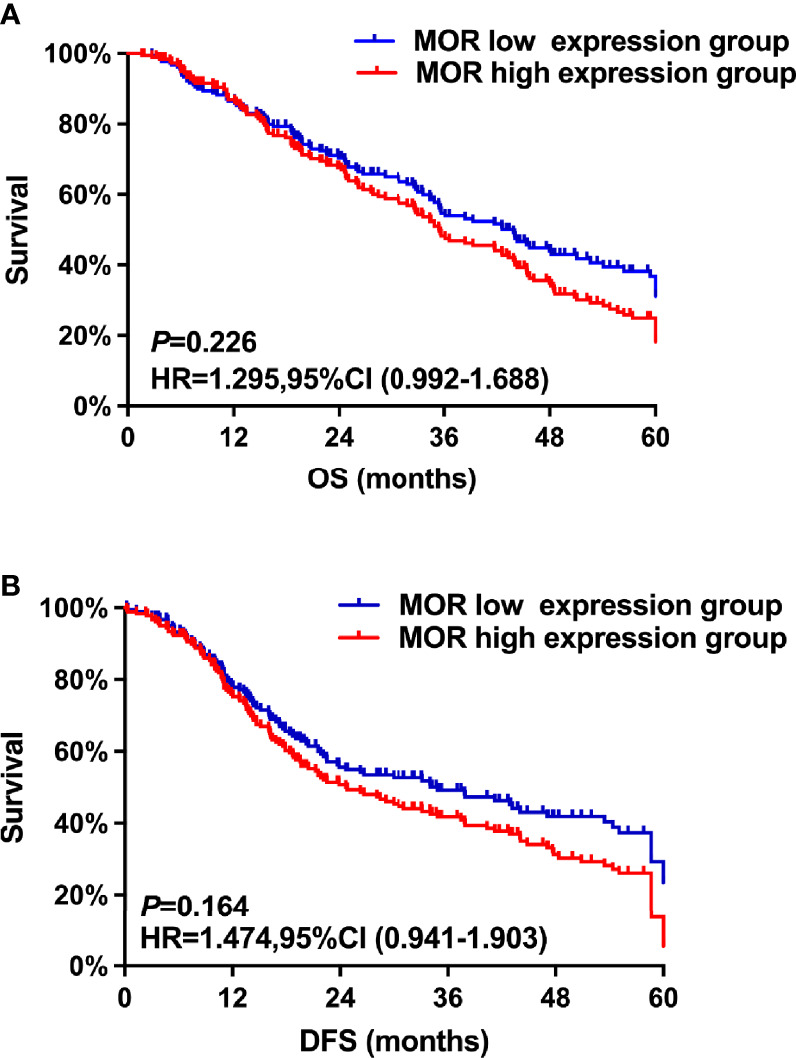
The study’s co-primary outcomes were **(A)** overall survival analysis based on MOR expression and **(B)** disease-free survival based on MOR expression.

The median follow-up time in all patients was 45.4 (43.2, 47.3) months. The Kaplan–Meier survival curves for the MOR high expression and MOR low expression are shown in **Figure 1**. There were no significant differences in OS rate at the first, third, and fifth years between the MOR high expression and MOR low expression groups (1-year OS: 82.9%, vs. 83.3%, 3-year OS: 57.8%, vs. 59.1%, and 5- year OS: 22.4% vs. 23.1%, respectively, **Figure 1A**). The univariate analysis indicated that the following covariates were significantly associated with worse OS: age, ASA physical status, CCI, non-serous histology, poor tumor differentiation, residual disease, surgical complexity, ascites, estimated blood loss, and no adjuvant chemotherapy (**Table S1**).

The multivariate analysis after propensity score matching demonstrated that non-serous histology (HR = 1.86, 95% CI: 1.32–2.38, P = 0.018), poor tumor differentiation (HR = 1.26, 95% CI: 1.13–2.73, P < 0.001), residual disease (HR = 1.46, 95% CI: 1.02–1.94, P = 0.023), and no adjuvant chemotherapy (HR = 1.36, 95% CI: 1.12–1.73, P = 0.026) were associated with worse OS (**Table 2**). A high MOR expression was not a predictor of worse OS (HR = 1.30, 95% CI: 0.99–1.69, P = 0.226).

**Table 2:** Multivariable Cox proportional of OS and DFS.

Variables	OS (before matching)		OS (after matching)		DFS (before matching)		DFS (after matching)	
	HR (95% CI)	*P*-value	HR (95% CI)	*P*-value	HR (95% CI)	*P*-value	HR (95% CI)	*P*-value
**Histologic diagnosis (non-serous histology)**	1.93 (1.22–2.98)	0.026	1.86 (1.32–2.38)	0.018	2.30 (1.62–2.92)	0.018	2.13 (1.74–2.88)	0.046
**Tumor differentiation (poor)**	1.44 (1.02–2.78)	0.011	1.26 (1.13–2.73)	<0.001	1.76 (1.62–2.88)	0.023	1.68 (1.42–2.75)	0.035
**FIGO stage (III–IV)**	1.58 (1.15–2.15)	<0.001	1.39 (1.22–1.88)	<0.001	1.63 (1.45–2.35)	<0.001	1.53 (1.48–2.28)	<0.001
**Residual disease (>1 cm)**	1.46 (1.23–1.58)	<0.001	1.46 (1.02–1.94)	0.023	1.83 (1.62–1.98)	0.026	1.76 (1.22–2.42)	<0.001
**Postop-chemotherapy (no)**	1.75 (1.41–1.62)	<0.001	1.36 (1.12–1.73)	0.026	2.54 (1.32–2.88)	<0.001	2.34 (1.12–2.63)	<0.001

OS, overall survival; DFS, disease-free survival.

Similarly, there were no significant differences in first-, third-, and fifth-year DFS rates between the MOR high-expression cohort and the MOR low-expression group of patients (1-year DFS: 77.3%, vs. 78.6%, 3-year DFS: 47.8%, vs. 48.3%, and 5- year DFS: 18.4% vs. 22.1%, respectively, **Figure 1B**). The univariate analysis indicates that the following covariates were significantly associated with worse OS: age, ASA, CCI, non-serous histology, poor tumor differentiation, residual disease, surgical complexity, ascites, estimated blood loss, and adjuvant chemotherapy (**Table S1**).

The multivariate analysis after propensity score matching indicated that non-serous histology (HR = 2.13, 95% CI: 1.74–2.88, P = 0.046), poor tumor differentiation (HR = 1.68, 95% CI: 1.42–2.75, P = 0.035), FIGO stage (HR = 1.53, 95% CI: 1.48–2.28, P < 0.001), residual disease (HR = 1.76, 95% CI: 1.22–2.42, P < 0.001), and no adjuvant chemotherapy (HR = 2.34, 95% CI: 1.12–2.63, P < 0.001) were associated with shorter DFS (**Table 2**). A high MOR expression was not a predictor of worse DFS (HR = 1.47, 95% CI: 0.94–1.90, P = 0.164).

### Secondary Outcomes

**Figure 2:**
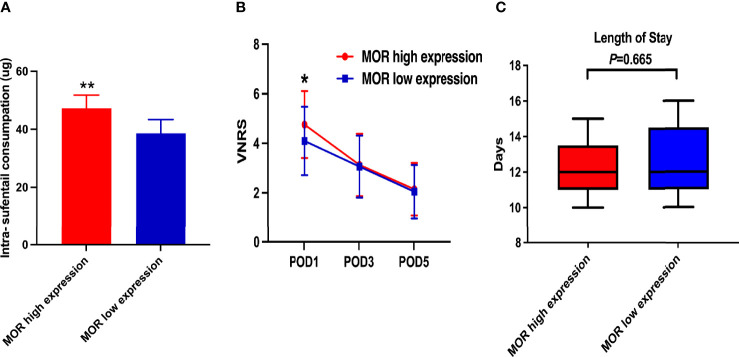
Secondary outcomes of the study. **(A)** Intraoperative sufentanil consumption according to MOR expression; **(B)** VNRS on POD1, POD3, and POD5 according to MOR expression; and **(C)** LOS according to MOR expression. MOR, mu-opioid receptor; VNRS, verbal numerical rating scale. *P < 0.05, **P < 0.01.

**Figure 3:**
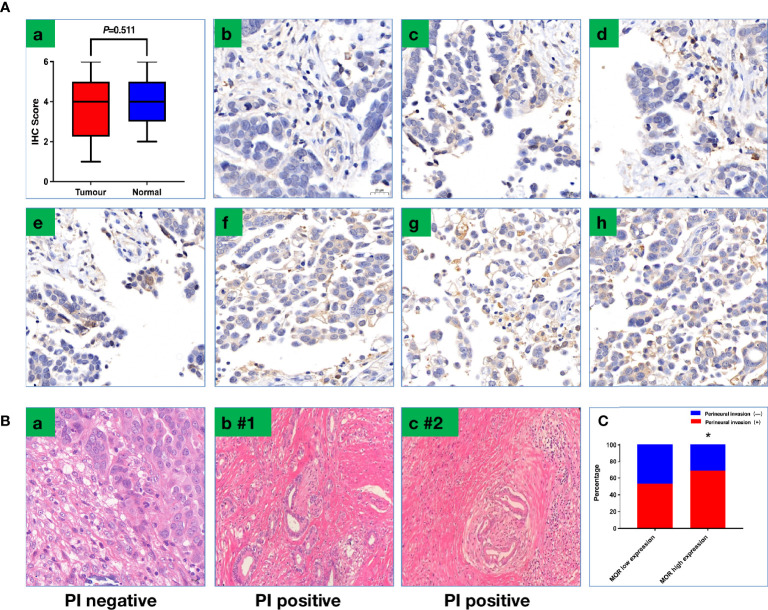
**(A)** Representative images of IHC to show scoring criteria and MOR expression. (a) MOR expression between tumor tissue and normal tissue; (b) score 0; (c) score 1; (d) score 2; (e) score 3; (f) score 4; (g) score 5; (h) score 6. **(B)** Representative image to show PNI; PNI was defined as cancer cells that invade the perineural spaces of surrounding nerves (a) PNI negative; (b,c) PNI-positive patients (#1–2). **(C)** PNI positive rate based on MOR expression. PNI, perineural invasion, *P < 0.05.

The mean intraoperative sufentanil consumption in the MOR high-expression group was significantly higher than in the MOR low-expression group (47.2 ± 4.6 vs. 38.6 ± 4.8, P < 0.001, **Figure 2A**). Pain intensity was higher on POD1 in the MOR high-expression cohort compared with the MOR low-expression group (4.76 ± 1.35 vs. 4.10 ± 1.38, P = 0.024, **Figure 2B**). The mean LOS in the MOR high-expression group was 12.7 (11.3, 13.8) days compared with 12.0 (11.4, 14.2) days in the MOR low-expression group (P = 0.665, **Figure 2C**). There were no differences in MOR expression between tumor and normal tissue (mean: 4.2 vs.4.4, P = 0.551, **Figure 3A**). Interestingly, we observed that a high level of MOR expression was associated with a significantly higher rate of PNI (68.9% vs. 53.4%, P = 0.037, **Figures 3B, C**).

## Discussion

In this study, we evaluated the association between MOR expression and ovarian cancer long-term outcomes in patients undergoing PDS. This study found that MOR expression did not significantly affect OS and DFS.

These findings parallel the results of two previous studies in pancreatic cancer (11, 13), indicating that MOR expression in pancreatic ductal adenocarcinoma (PDAC) patients was not associated with worse OS and DFS. Diaz-Cambronero et al. also observed that high levels of MOR expression did not significantly impact the survival of patients with colorectal cancer (12). In contrast, our previous study found that an increased MOR expression was associated with reduced DFS and OS in subjects with laryngeal squamous cell carcinoma (9). At the *in vitro* level, MOR was found to promote and support tumor growth in lung cancer and hepatocellular carcinoma (18, 19). Furthermore, Gorur et al. observed that downregulating the MOR expression inhibited aggressive cell behaviors in squamous cell carcinoma of the head and neck (20). Fiegl et al. found no benefit of D,L-methadone (opioid agonist) as an adjuvant chemosensitizing anticancer drug in ovarian cancers (21). In their *in vitro* studies, there were no direct anticancer effects found in 2D and 3D cell culture experiments. In addition, the authors observed somewhat contrary results from the 3D cell culture model in which D,L-methadone could either enhance ovarian cancer cell proliferation or counteract the therapeutic effects of cisplatin (22). It is difficult to compare our results with these *in vitro* studies (18–21). The possible reason to explain the discrepancy from *in vitro* studies is bias and confounding owing to unknown and unmeasured variables that might have an impact on the clinical survival outcomes (22–24). Secondly, the difference in the type of cancer, stage of cancer, and the extent of surgical type all may account for the varied effects of MOR and survival outcomes (22–24). Thirdly, different-opioid consumption could have different effects on tumor growth and clinical survival outcomes (25). Our study also showed that tumor differentiation, FIGO stage, residual disease, ascites, and intraoperative and adjuvant chemotherapy were predictors of poor outcomes, as previously reported in other studies (26–29).

Interestingly, we observed that patients with a high expression of MOR also required higher dosages of sufentanil. At least three previous studies reported similar findings in patients with prostate, laryngeal, and pancreatic cancers (9, 11, 13). However, the mechanism by which a higher expression of MOR in tumor specimens is associated with increased consumption of intraoperative opioids is still unclear. PNI is associated with pain and predicts worse outcomes in ovarian cancers (30–32). We can speculate that high levels of tumoral MOR can promote neuronal sensitization in response to an inflammatory tumor microenvironment (33). This is supported by the fact that patients with a higher expression of MOR also had higher pain levels on POD1. Alternatively, elevated concentrations of locally released endorphins in patients with pain could be responsible for a high rate of perineural invasion (34).

In this study, we evaluated the association between MOR expression and survival outcomes in ovarian cancers. Our study has limitations as follows. Firstly, the retrospective design of the study may introduce bias and the negative result that MOR is not associated with OS or DFS could be due to being underpowered. Secondly, while our study shows no association between MOR expression level and outcomes, this does not enable any conclusions regarding the effect of opioids (intraop etc.) on these outcomes. Thirdly, we did not perform a subgroup survival analysis of opioid consumption and MOR expression [high opioid consumption and high MOR expression (HOHM), high opioid consumption and low MOR expression (HOLM), low opioid consumption and high MOR expression (LOHM), low opioid consumption and low MOR expression (LOLM)] since not only MOR expression but further opioid exposure could have impact on the survival outcomes. Last, we did not investigate the mechanism implicated in tumoral MOR expression and perineural invasion.

In conclusion, MOR expression was not associated with OS or DFS in ovarian cancer patients. Our results indicated a high level of MOR expression associated with perineural invasion in ovarian cancers.

## Data Availability Statement

The datasets presented in this study can be found in online repositories. The names of the repository/repositories and accession number(s) can be found in the article/**Supplementary Material**.

## Ethics Statement

The studies involving human participants were reviewed and approved by the Ethics Committee of Fudan University (No. 20200206). The patients/participants provided their written informed consent to participate in this study.

## Author Contributions

HZ, and CM conceived and designed the study. HZ, MQ, ZS, and YW, WX, MS, and TL collected the data. HZ, XZ, MS, and WC interpreted and analyzed the data. HZ were the major contributors in writing the manuscript. HZ, and CM reviewed and edited the manuscript. All authors contributed to the article and approved the submitted version.

## Funding

This research was supported by the National Natural Science Foundation of China (Nos. 82102253, 81871591), Clinical Research Plan of SHDC (Nos. SHDC2020CR1005A, SHDC2020CR4064), Natural Science Foundation of Shanghai (No. 21ZR1413400), Shanghai Sailing Program (No. 21YF1406800), the Shanghai Municipal 2021 “Science and Technology Innovation Action Plan” (Nos. 21JC1401400, 21S31902600), and 2019 Fudan University Zhuo-Xue Project (No. JIF159607).

## Conflict of Interest

The authors declare that the research was conducted in the absence of any commercial or financial relationships that could be construed as a potential conflict of interest.

## Publisher’s Note

All claims expressed in this article are solely those of the authors and do not necessarily represent those of their affiliated organizations, or those of the publisher, the editors and the reviewers. Any product that may be evaluated in this article, or claim that may be made by its manufacturer, is not guaranteed or endorsed by the publisher.
