# Supplementary material for: Association of Mu-Opioid Receptor Expression With Long-Term Survival and Perineural Nerve Invasion in Patients Undergoing Surgery for Ovarian Cancer
Source: Front Oncol. 2022 Jul 7;12:927262. doi: 10.3389/fonc.2022.927262 (PMC9302566; doi:10.3389/fonc.2022.927262)
Supplement: Supplementary file 1 [file Table_1.doc]

**Table S1**. Univariate analysis of OS and DFS

| **Variables** | **OS** | | **DFS** | |
| --- | --- | --- | --- | --- |
|  | HR (95% CI) | *P*-value | HR (95% CI) | *P*-value |
| **Age (years)** | 1.03(1.01-1.15) | 0.024 | 1.14(1.08-1.36) | 0.024 |
| **BMI (kg/m2）** | 1.01(0.95-1.05) | 0.265 | 1.06(1.00-1.27) | 0.452 |
| **ASA score (III-IV)** | 1.33(1.24-1.83) | 0.036 | 1.43(1.16-1.58) | 0.032 |
| **CCI (>2)** | 1.25(1.19-1.54) | 0.045 | 1.38(1.11-1.62) | 0.023 |
| **Histologic diagnosis (Non-serous histology)** | 1.37(1.25-1.68) | <0.001 | 1.47(1.32-1.73) | <0.001 |
| **Tumor differentiation (poor)** | 1.47(1.22-1.78) | 0.015 | 1.66(1.26-2.13) | 0.032 |
| **Residual disease (>1cm)** | 1.92(1.55-2.36) | 0.018 | 1.98(1.36-2.46) | 0.025 |
| **Surgical complexity** | 1.25(1.18-1.76) | 0.025 | 1.46(1.32-1.96) | 0.034 |
| **Ascites (ml)** | 1.66(1.42-2.18) | 0.033 | 1.75(1.30-2.28) | 0.012 |
| **Estimated blood loss (ml)** | 1.32(1.15-1.52) | 0.035 | 1.50(1.33-1.72) | 0.024 |
| **Postop-Chemotherapy (no)** | 1.97(1.44-2.76) | <0.001 | 2.36(1.72-2.52) | <0.001 |

Abbreviations: BMI: Body Mass Index; ASA: American Society of Anesthesiologists score; CCI: Charlson Comorbidity Index; OS: Overall Survival; DFS: Disease free Survival
